# Network Evolution: Rewiring and Signatures of Conservation in Signaling

**DOI:** 10.1371/journal.pcbi.1002411

**Published:** 2012-03-15

**Authors:** Mark G. F. Sun, Martin Sikora, Michael Costanzo, Charles Boone, Philip M. Kim

**Affiliations:** 1 Department of Computer Science, University of Toronto, Toronto, Canada; 2 Banting and Best Department of Medical Research, University of Toronto, Toronto, Canada; 3 Terrence Donnelly Centre for Cellular and Biomolecular Research, University of Toronto, Toronto, Canada; 4 Institut de Biologia Evolutiva (UPF-CSIC), CEXS-UPF-PRBB, Barcelona, Spain; 5 Department of Molecular Genetics, University of Toronto, Toronto, Canada; University of Zurich and Swiss Institute of Bioinformatics, Switzerland

## Abstract

The analysis of network evolution has been hampered by limited availability of protein interaction data for different organisms. In this study, we investigate evolutionary mechanisms in Src Homology 3 (SH3) domain and kinase interaction networks using high-resolution specificity profiles. We constructed and examined networks for 23 fungal species ranging from *Saccharomyces cerevisiae* to *Schizosaccharomyces pombe*. We quantify rates of different rewiring mechanisms and show that interaction change through binding site evolution is faster than through gene gain or loss. We found that SH3 interactions evolve swiftly, at rates similar to those found in phosphoregulation evolution. Importantly, we show that interaction changes are sufficiently rapid to exhibit saturation phenomena at the observed timescales. Finally, focusing on the SH3 interaction network, we observe extensive clustering of binding sites on target proteins by SH3 domains and a strong correlation between the number of domains that bind a target protein (target in-degree) and interaction conservation. The relationship between in-degree and interaction conservation is driven by two different effects, namely the number of clusters that correspond to interaction interfaces and the number of domains that bind to each cluster leads to sequence specific conservation, which in turn results in interaction conservation. In summary, we uncover several network evolution mechanisms likely to generalize across peptide recognition modules.

## Author Summary

Protein interaction networks control virtually all cellular processes. The rules governing their evolution have remained elusive, as comprehensive protein interaction data is available for only a small number of very distant species, making evolutionary network studies difficult. Here we attempt to overcome this limitation by computationally constructing protein interaction networks for 23 relatively tightly spaced yeast species. We focus on networks consisting of kinase and peptide binding domain interactions, which play central roles in signaling pathways. These networks enable us to investigate evolutionary network mechanisms. We are able, for the first time, to accurately quantify the contribution of different rewiring mechanisms. Interaction change appears to be mainly accomplished through binding site evolution rather than through gene gain or loss. This is in contrast to other evolutionary processes, where gene duplication or deletion is a major driving factor. Moreover, our analysis reveals that interaction changes are very fast – fast enough that the number of changes saturates, i.e., the actual rate of change has been strongly underestimated in previous studies. Our analysis also reveals different mechanisms by which certain interactions are conserved throughout evolution. Our results likely transfer to other species and networks, and will benefit future evolutionary studies of signaling pathways.

## Introduction

Peptide recognition modules (PRMs) and kinase domains bind short linear peptide motifs on their protein binding partners and are integral members of many signaling pathways [1]–[3]. PRM members include the SH3 (Src homology 3), SH2 (Src homology 2), and PDZ (PSD-95/Discs-large/ZO-1) domains [1]. In this study, we focus on the SH3 domain, a small (∼60 amino acids) domain, implicated in crucial regulatory processes such as signal transduction, cytoskeleton organization, and cell polarization [4], [5]. SH3 domains typically bind short proline rich peptides containing a PxxP binding motif [5]. Initial structural analysis revealed two main binding classes, although variations to these canonical SH3 binding motifs have also been discovered.

Experimental identification of the short peptide binding motifs recognized by PRMs has been performed using a number of methods, such as synthetic peptide arrays (SPOT), oriented peptide array libraries (OPAL), protein domain microarrays, and phage display [1], [6]–[10]. Binding specificity maps have been generated for *Saccharomyces cerevisiae* SH3 and kinase domains using phage display and combinatorial peptide library screening approaches, respectively [6], [11]. Domain binding specificities from these experimental methods are captured in position weight matrices (PWMs) enabling comprehensive and high confidence predictions of physical interactions involving SH3 and kinase domains. The high accuracy of these PWM predictions has been demonstrated in their ability to recapitulate interactions derived from orthogonal experimental methods such as yeast two-hybrid [6].

Interaction network studies have uncovered network properties such as scale-free and hierarchical topologies [12], resulting in the development of models describing protein interaction network evolution [12]–[15]. Network rewiring rates for protein interaction networks have also been established for proteins in *S. cerevisiae* that have paralogs [16], model eukaryotic protein interaction networks [17], and yeast regulatory networks [18]–[20]. A global comparative analysis on network rewiring from existing experimental datasets has suggested that regulatory networks are among the fastest evolving biological networks [21]. However, these comparative studies are hampered by two problems: The analyzed networks are often incomplete and the species examined are highly diverged. An obvious problem is that interactions in species similar to the model organisms (such as yeast or worm) are usually inferred by means of orthology mapping, which prohibits any kind of evolutionary analysis based on them [22], [23]. Unlike these mapping methods, predicting interactions via PWMs enables generation of interaction networks in a species-independent manner. This permits a more accurate means to identify both conserved and diverged interactions, as interactions are determined by a domain's binding specificity and not by orthologous protein pairs, thus enabling the evaluation of different evolutionary mechanisms that give rise to the observed rates of network rewiring.

In this study, we use the aforementioned high resolution *S. cerevisiae* SH3 and kinase specificity maps [6], [11] to computationally predict high confidence SH3 and kinase interaction networks for 23 species belonging to the Ascomycota phylum of the Fungal kingdom, representing over 300 million years of evolution [24]. We quantify network evolution rates for different network rewiring mechanisms and compare them to other network evolutionary rates [18]. Furthermore, we show that the rate of network rewiring reaches saturation due to the rapid rate of interaction change. Moreover, we uncover interaction conservation patterns related to multiple SH3 domains binding the same proline rich region on a protein binding partner. Finally, we show motif specific sequence conservation translates to the conservation of interactions.

## Results/Discussion

### SH3 and kinase interaction networks for 23 fungal species

**Figure 1:**
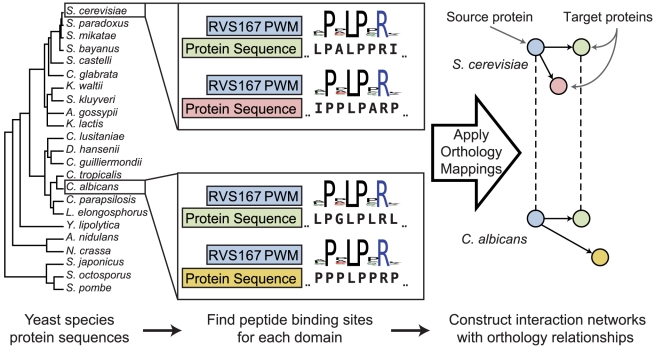
Generating SH3 and kinase interaction networks schematic. 23 yeast proteomes were scanned with 30 SH3 domain and 63 kinase domain position weight matrices (PWMs). High scoring interactions are selected and merged together on a per species basis to form SH3 and kinase interaction networks. In order to compare the different species' networks, orthology mappings were used [25]. Vertices (nodes), solid directed arrows, and dashed undirected lines represent proteins, protein interactions, and orthology assignments respectively. Blue nodes are proteins with an SH3 or kinase domain and any other colored node is a target protein of either a SH3 or kinase domain. Nodes of the same color but in different species are orthologs.

Using position weight matrix (PWM) profiles derived from *S. cerevisiae* phage display experiments and combinatorial peptide library screens [6], [11], we constructed SH3 and kinase interaction networks in 23 different fungal species spanning over 300 million years of evolution (Figure 1, Materials and Methods). Note that our methodology does not rely on sequence homology to predict interactions in different species, enabling the identification of species-specific interactions through binding profiles. Sequence homology is used only in the comparison of interaction networks between different species. The SH3 predicted network, using 30 PWMs from *S. cerevisiae* resulted in ∼800 interactions and ∼400 unique proteins for each of the 23 yeast species. Likewise, the kinase network, using 63 PWMs, resulted in ∼1800 interactions and ∼450 unique proteins. Parameters to create the networks were selected using the area under the receiver operator curve (AUROC) and the Matthews correlation coefficient metrics (Materials and Methods). In Figure S1 we provide estimates over a range of true positive and true negative ratios.

To compare the constructed networks and infer interaction conservation among the different yeast species, orthology assignments provided by Wapinski and co-workers were used to establish orthology relationships for all proteins in the networks (Materials and Methods) [25]. We further ensured that orthologs to the *S. cerevisiae* SH3 and kinase proteins contained the particular SH3 and kinase domains, respectively. Here, we assume domain binding specificities found in *S. cerevisiae* to be similar for orthologous proteins, even for distant species. While this is a critical assumption, five different observations suggest it is reasonable for the proteins analyzed. First, we examined paralogous domains in *S. cerevisiae* since they tend to be under weaker purifying selection than non-duplicated genes and are likely to diverge at faster rates than proteins in different species [26]. We find paralogous SH3 proteins to have very high PWM similarity, especially considering those above 80% amino acid sequence identity (Figure S2). Second, we show orthologs and paralogs share the same amino acids at similar, presumably binding determining, positions in a multiple sequence alignment (Figure S3). Third, SH3 domain crystal structures reveal contact amino acids to a bound ligand are highly conserved in orthologs (Figure S4). Fourth, orthologs to *S. cerevisiae* SH3 and kinase domains exhibit a high degree of amino acid identity (Figure S5A and S6). Fifth and finally, for many following analysis, we use two species sets: one where we use all 23 species and a restricted set, where we use orthologs with an amino acid sequence identity greater than 80% (for which binding specificity is almost guaranteed to be conserved). In all cases, we observe the same results.

### SH3 and kinase interaction network structure and topology is conserved

**Figure 2:**
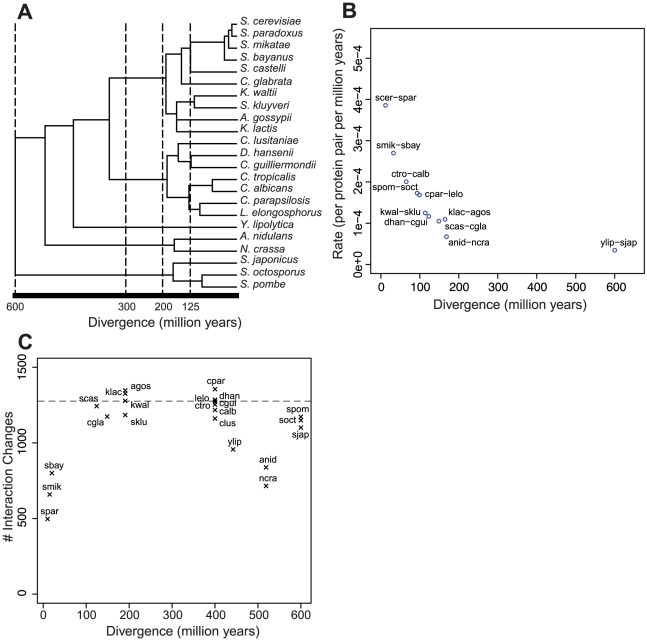
Rates of interaction change. Divergence distances are taken with respect to the last common ancestor. A) The canonical phylogeny based on protein sequences of common genes found in all 23 species (Materials and Methods). B) Rates of SH3 interaction change calculated such that no branch is shared in the canonical phylogenetic tree versus divergence in millions of years. C) The number of SH3 interaction changes with respect to *S. cerevisiae* versus divergence in millions of years. Saturation is reached within ∼200 million years of divergence.

To assess the similarity of the fungal networks with each other, we created phylogenetic trees from the orthology mapped interaction networks, based on the number of conserved interactions between species (Materials and Methods). Importantly, we found that the phylogenetic trees derived from the predicted SH3 and kinase interactions () are remarkably similar to the canonical protein sequence-based phylogeny (Figure 2A), suggesting that the interaction networks share similar evolutionary properties as genome sequences [27]. While we observe similar phylogenetic trees for the fungal species, analysis spanning the 3 domains of life revealed topological differences between the metabolic pathway and sequence based phylogenetic trees, representing many more years of evolution [28]–[30]. Despite the phylogenetic similarities for the fungal species, only 5 SH3 interactions are conserved across all 23 yeast species, translating to ∼1% of all *S. cerevisiae* SH3 interactions. For kinases, not a single interaction is conserved across all kinase interaction networks given the defined thresholds and orthology mappings. The limited number of globally conserved interactions indicates that phylogenetic similarities are due to conservation of the network structure and topology rather than individual interactions between orthologs.

### Quantification of evolutionary changes in the SH3 and kinase interaction networks

The observed lack of conservation of specific interactions may be attributed to two main evolutionary mechanisms. First, an interaction can be lost or gained but both the binding and target proteins are evolutionary retained, which we refer to as “interaction rewiring” (i.e., orthologs exist for the associated genes of the binding and target proteins). Second, the binding or target protein itself can be gained or lost (i.e. through a gene duplication), which we refer to as a “protein change”. To examine interaction differences observed in these networks, we quantified rates of interaction rewiring and protein change in both the SH3 and kinase networks and compared them to previously reported rates for other types of interaction networks.

To attain a rate of interaction rewiring, we need to ascertain the number of interaction changes between two species as well as their divergence time (see Materials and Methods). We find that the interaction rewiring rate decreases sharply as the divergence distance increases for the SH3 and kinase interaction networks (Figure 2B, Figure S8A) in addition to being slower than in random networks (p-value<0.001, Figure S9). This suggests that to obtain an accurate rewiring rate one has to use closely related species, as the estimated rewiring rates are dependent on divergence distance and hence the selected reference species. For example, the rates of interaction rewiring in the SH3 network between closely related species 1) *S. cerevisiae* and *S. paradoxus* and between distant species 2) *S. cerevisiae* and *S. pombe* are 3.86×10^−4^ and 4.01×10^−5^ interaction rewirings per protein pair per million years respectively. Indeed, using the nearest evolutionary species as a reference, *S. octosporus*, for *S. pombe* results in an over 4 times increase to 1.85×10^−4^ interaction rewiring per protein per million years (Table S1). Intuitively, this phenomenon may be explained by a saturation of interaction changes at longer evolutionary distances. A similar result is observed when selecting SH3 domains above a range of amino acid identity thresholds (Table S2, Figure S10), indicating that our assumption of orthologs retaining similar specificities is reasonable, as noted above.

To investigate this saturation effect, we examined the absolute number of interaction changes with respect to divergence time. Importantly, we find the number of SH3 and kinase interaction rewiring events is sufficiently rapid to reach saturation in about 200 million years of divergence since the last common ancestor (Figure 2C, Figure S8B). This saturation effect is analogous to the saturation of neutral sequence substitutions in the comparison of highly divergent sequences resulting in a biased dN/dS ratio, due to the inability to observe multiple substitutions at the same nucleotide positions thus resulting in a deflated dS value. The observed decrease in interaction rewiring rate is dominated by divergence distance and possibly reflects the inability to observe multiple rewirings of the same interaction, as the loss followed by a gain of the same interaction appears as a conserved interaction (Table S1). Previous studies that quantified evolutionary rates for network changes [18], [19] used relatively distant species for comparison and were hence likely hampered by this issue. Thus we expect that they have underestimated the true evolutionary rate.

Similar to interaction rewiring, the rate of protein change is also dependent on the selected reference species. Using the nearest species to calculate rates in the SH3 network, the average rate of protein change is 1.95×10^−5^ protein changes per protein pair per million years while the average rate of interaction rewiring is 1.61×10^−4^ interaction rewiring per protein pair per million years (Table S1 and S3). An almost 10-fold difference between rates in interaction rewiring and protein change suggests that interaction rewiring is the primary mechanism for determining the overall rate of interaction change in the SH3 interaction network. A similar result is observed in the kinase network (Table S2 and S4). SH3 and kinase signaling interactions are known to evolve more rapidly than metabolic and other protein interactions [17], [21], due to the short protein binding surface area forming the interaction interface. Additionally, the time scale to observe the saturation phenomena in these networks is significantly smaller than the time scales used in previous comparison studies [17], [21]–[23]. Here we find the dominant mechanism for interaction change, at the observed time scale, is by interaction rewiring for these signaling networks. This is reminiscent of the study by Zhong *et al.* who found the mechanisms of interaction rewiring and protein change corresponded to distinct mutation events. They further found single genes associated with multiple diseases could be explained by interaction rewiring (ie. network perturbation) [31]. Thus the rapid rewiring rate exhibited in the SH3 and kinase networks conceivably enables the discovery of new functionality while maintaining the same gene repertoire.

### Rates of interaction change in the SH3 and transcription factor networks are similar to rates found in the kinase network

A previous study examined the rate of interaction change (i.e. the combination of interaction rewiring and protein change rates) associated with phosphosite and transcription factor regulatory networks [18]. We therefore compared rates for these networks to rates for our SH3 and kinase interaction networks. To provide an unbiased comparison, by compensating for the aforementioned saturation phenomenon, we computed interaction change rates of our kinase network using *S. cerevisae* as the sole reference strain instead of the closest related species and observed similar rates between the two networks. Specifically, interactions in the *C. albicans* and *S. pombe* phosphosite network were reported to change at a rate of 1.09×10^−5^ and 1.24×10^−5^ interaction changes per protein pair per million years, respectively after adjusting to our orthology mappings [18] (Table S5). Importantly, we found that interaction changes in the *C. albicans* and *S. pombe* kinase networks occur at a comparable rate, 3.68×10^−5^ and 3.89×10^−5^ interaction changes per protein pair per million years respectively (Table S2 and S4), when *S. cerevisiae* was used as the reference species. Considering that our computational approach is likely to contain some false positives and false negatives, the calculated rates may be overestimated. Thus, the close agreement between our findings and the Beltrao *et al* phosphosite network study supports the validity of our computational approach.

We next quantified rates in the *C. albicans* and *S. pombe* SH3 networks and found that they change at a rate of 5.90×10^−5^ and 5.47×10^−5^ interaction changes per protein pair per million years, respectively (Table S1 and S3). Interestingly, interaction changes in the SH3 networks occur at similar rates compared to interaction changes in the kinase and phosphosite networks.

Rates for the transcription factor-DNA (TF-DNA) interactions have also been deduced for *S. mikatae* and *S. bayanus* regulatory networks using *S. cerevisiae* as a reference species. We found that the rates of interaction change associated with these regulatory networks (1.02×10^−3^ and 5.91×10^−4^ interaction changes per transcription factor-gene pair per million years for *S. mikatae* and *S. bayanus*, respectively) (Table S6) [18] are similar to the SH3 interaction network rates of interaction change for the same species (4.82×10^−4^ and 4.24×10^−4^ interaction changes per protein pair per million years for *S. mikatae* and *S. bayanus* respectively) (Table 1). Given enhancer regions diverge at a fast rate [32], [33], transcription factor interactions are expected to change rapidly.

**Table 1:** Quantification of the two mechanisms of yeast SH3 interaction change: 1) interaction rewiring, and 2) protein change.

Species	SH3 domain orthologs	Orthologs	Changed interactions	Divergence Time (My)	SH3 interaction change (per protein pair per My)
*S. paradoxus*	21	5096	497	10	4.64×10−4/3.86×10−4
			61		5.70×10−5/4.74×10−5
*S. mikatae*	21	4913	659	15	4.26×10−4/3.05×10−4
			88		5.69×10−4/4.08×10−5
*S. bayanus*	21	4996	800	20	3.81×10−4/2.36×10−4
			91		4.34×10−5/2.68×10−5
*C. albicans*	17	3982	1217	400	4.49×10−5/2.01×10−4
			381		1.41×10−5/1.92×10−5
*S. pombe*	15	3247	1172	600	4.01×10−5/1.85×10−4
			426		1.46×10−5/4.42×10−6

Rows including the species name contain the interaction rate for the SH3 interaction change denoted by 1) above, while the row immediately below illustrates the rate for the second type of interaction change. Rates before and after the backslash were calculated respectively by using *S. cerevisiae* and the closest species to the species in question from the gene derived phylogenetic tree as the reference species. Divergence time is taken with respect to the last common ancestor in millions of years.

Here we find that different peptide recognition domain networks evolve at different rates, all of which are faster by an order of magnitude than the rate of change of protein-protein interaction networks [21]. This is the case even when considering the estimated error in the rate of network rewiring within the SH3 interaction network between the evolutionary closest species of *S.cerevisiae* and *S. paradoxus* (estimated at 1.7×10^−4^ interaction changes per protein pair per million years). Given the rate at which the signaling and regulatory networks rewire, it is tempting to speculate that the ability to rapidly reorganize their structure is a mechanism for swift adaptation to selective constraints while minimizing disruption to a core network responsible for basic cellular functionality.

### Global trends in interaction conservation

**Figure 3:**
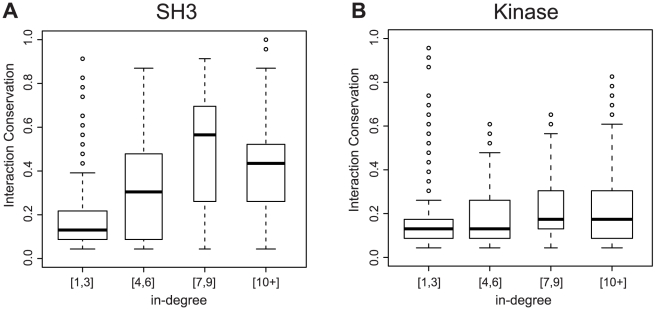
Significant correlations exist between interaction conservation and the number of interaction partners of a target protein in the *S. cerevisiae* SH3 and kinase interaction networks. A) A strong correlation between interaction conservation and the number of interaction partners of a target protein for the SH3 protein interaction network (ρ = 0.466, p-value<2.2×10^−16^) and B) for the kinase protein interaction network (ρ = 0.337, p-value<2.2×10^−16^).

The rates above highlight the plastic nature of SH3 and kinase interaction changes which in addition to the lack of a significant correlation between a SH3 PWM's entropy and the rate of interaction rewiring (ρ = −0.067, p-value = 0.724, Figure S11) suggest detecting conserved interaction signals to be difficult. Interestingly, we readily observed global trends of network conservation. Using *S. cerevisiae* as the reference species, we found a significant correlation between the number of domains that bind a target protein and the degree to with interactions are conserved, for both the SH3 (ρ = 0.466, p-value<2.2×10^−16^) and kinase (ρ = 0.337, p-value<2.2×10^−16^) interaction networks (Figure 3A and 3B). These correlations explain 16% and 10% of the variance, respectively, where interaction conservation is the fraction of species retaining an interaction found in *S. cerevisiae*. This suggests targeted proteins may retain interactions by maintaining many interaction partners (ie. the target protein has a high in-degree).

**Figure 4:**
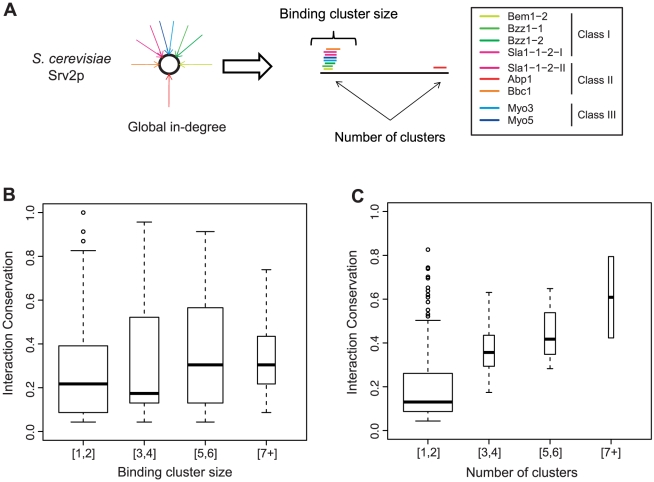
Binding cluster formation and existence of a significant correlation between interaction conservation and both binding cluster size and the number of binding site clusters. A) Global protein in-degree decomposition into amino acid resolution binding demonstrates the formation of binding site clusters. A segment of the *S. cerevisiae* protein Srv2p is shown demonstrating binding cluster formation B) A strong and significant correlation between interaction conservation and binding cluster size exists (ρ = 0.192, p-value = 4.67×10^−6^). C) The correlation between interaction conservation and the number of clusters is found to have an even stronger correlation with the number of clusters found on a protein for the SH3 interaction network (ρ = 0.461, p-value = 5.85×10^−12^).

To identify mechanisms giving rise to the above correlation, we use the position specific binding information provided by the PWMs to determine the exact region bound by a domain on a target protein (Materials and Methods). We found that high in-degree target proteins have peptide regions bound by multiple SH3 domains, forming binding site clusters primarily in proline rich regions (Figure cluster 4A). Binding site cluster formation may be attributed to two modes: 1) binding by the same SH3 specificity class and 2) binding by multiple SH3 specificity classes that share a common PXXP core, where X is any amino acid. As an example, Srv2p contains a multiclass cluster composed of class I, II, and III SH3 domain binders (Figure 4A). While we observe the formation of clusters, two proteins cannot simultaneously occupy the same binding site, thus multiple binding domains forming a cluster competitively bind for the target binding site.

Having identified cluster formations at SH3 target binding sites, we explored the relationship between the size of the binding site clusters and interaction conservation. A significant correlation was found between cluster size and interaction conservation (ρ = 0.192, p-value = 4.67×10^−6^) (Figure 4B), though not as significant as the correlation found at the global level of protein interactions between the number of interacting SH3 domains and interaction conservation. Interestingly, cluster sizes greater than 7 fail to exhibit the same degree of interaction conservation as proteins whose in-degree are of the same magnitude. Since proteins with many interacting SH3 domains may contain multiple clusters, this suggests the number of binding clusters may play a role in determining an interaction's conservation degree. Pursuing this observation, we find a significant correlation between the number of binding site clusters and interaction conservation (ρ = 0.461, p-value = 5.85×10^−12^) (Figure 4C). This is in agreement with previous studies suggesting that the amount of a protein participating in interactions is more conserved [34], [35]. The existence of disjoint clusters is analogous to the existence of several different interaction interfaces participating in a complex formation, which is most likely driving the correlation with interaction conservation. Similar observations are found within the kinase interaction network where correlations between interaction conservation and both binding site cluster size (ρ = 0.210, p-value = 1.40×10^−14^) and the number of clusters (ρ = 0.296, p-value = 6.48×10^−10^) are found (Figure S12).

### Sequence specific conservation relates to interaction conservation

Cluster formation at binding sites suggests that the sequence could be evolutionary constrained to preserve recognition by multiple binding domains. To investigate the relationship between sequence conservation and interaction conservation we measured the binding site divergence using the AL2CO algorithm (Materials and Methods) [36] and observe a significant correlation between sequence conservation and binding cluster size (ρ = 0.241, p-value = 2.80×10^−5^), indicating the existence of selective pressure to maintain sequence conservation due to multiple interacting partners. However, the relative contribution of different target binding site amino acids to a domain's binding specificity varies wildly. Consider the binding specificity of a class I SH3 domain with the consensus sequence RXXPXXP, where X is any amino acid. The first, middle, and last positions of the binding site are constrained to specific anchor amino acids, whereas the other positions are free to evolve to any other amino acid. Thus from the perspective of the class I domain, a binding site is conserved when the 3 anchor amino acids are present, while the other amino acids are free to evolve.

To measure the conservation of a target peptide sequence relative to a SH3 domain's binding specificity we developed a new metric. Using *S. cerevisiae* as the reference species, for an observed target binding site, we first calculate its PWM score and the PWM score for all orthologous proteins. The difference between a species' PWM score to that of the *S. cerevisiae* ortholog indicates the relative amount the target site has evolved. To provide a summary metric, PWM scores for each ortholog are weighted by their divergence distance from the *S. cerevisiae* ortholog (Materials and Methods). While many previous studies sought to identify conserved protein interactions [22], [23], here we show that this metric enables identification of both conserved and evolving interactions. Conserved interactions have values near zero as is the case for Srv2p which contains a highly conserved binding cluster of size 6. Evolutionary changing interactions bind evolving target binding sites identified by negative values, larger magnitudes indicate recently acquired target binding sites. One example of an evolved target binding site is found on Ubp7p in a cluster size of 5. The metric captures the evolving binding site, while only weak sequence conservation is observed. Not surprisingly, a significant correlation between the metric and interaction conservation (ρ = 0.425, p-value<2.2×10^−16^) is observed, highlighting the metric's ability to capture sequence conservation relevant to interaction conservation. In line with this, we find a significant correlation between the metric and binding site cluster size (ρ = 0.271, p-value = 2.41×10^−6^). This result demonstrates that binding specificity from multiple domains indeed places evolutionary constraint on the target binding site sequence.

In conclusion, we use the specificity profiles of a common signaling domain to generate a model network for the fungal clade spanning a wide range of evolutionary distances. In this manner we overcome the difficulty of comparing interaction networks of very divergent model organisms derived from a limited amount of experimental interaction data. We draw a number of conclusions that are biologically important: First, we find that the major driver of evolution of signaling pathways is interaction change and not gene duplication or loss. Second, we find network interaction changes are so rapid that they swiftly saturate, a phenomenon future studies will need to consider. Finally, we find signatures of network conservation and propose associated mechanisms. We expect our results can be generalized to many other signaling domains, such as SH2, WW, and PDZ domains, since they are affected by the same fundamental evolutionary processes.

## Materials and Methods

### Collection of proteomes and domain specificity map data

Genomic and proteomic data for 23 fungal species from the Ascomycota phylum were obtained from a variety of sources [37]–[43]. The same genomic and proteomic datasets were selected as those used to generate the orthology group assignments by Wapinski *et al.* [25], but extend to 23 fungal species which is available on their website as version 1.1 (retrieved August 2009).

30 SH3 position weight matrices (PWMs) for 25 of 27 *S. cerevisiae* SH3 domains were obtained from Tonikian *et al.* [6]. Kinase PWMs were obtained from Mok *et al.* for 61 of 122 *S. cerevisiae* kinase proteins, for a total of 63 kinase PWM classes [11]. SH3 PWMs were constructed by aligning peptide sequences derived from phage display to create amino acid frequencies for each ligand position. Kinase PWMs are based on intensity signal ratios for each amino acid at every ligand position from peptide library screens.

### Binding target site prediction using MOTIPS at high accuracy

Identifying putative target binding sites for each SH3 and kinase domain was determined using the Motif Analysis Pipeline (MOTIPS) [44], a method similar to ScanSite [45]. Binding target sites were identified for the 23 yeast species using the *S. cerevisiae* SH3 and kinase domain PWM specificity classes (see above). Here we use the term target binding site to refer to a peptide target prediction corresponding to a PWM class. Given a PWM, the binding target site score is defined by
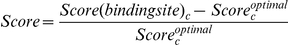
where *c* is the PWM class, 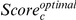 is the optimal binding score for the PWM class, and 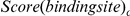 is defined below.
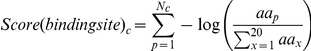
where *N_c_* is the length of the target binding site for class *c*, *p* is the position of an amino acid within the target binding site, and *aa* is an amino acid's entropic adjusted “count” [46] at a given position in the PWM. Each predicted binding target site is further associated with parameters of disorder (lack of tertiary structure) and surface solvent accessibility respectively computed by disopred2 and sable [47], [48] to provide additional features to enhance target binding site predictions.

Parameter selection was performed using the area under the receiver operator curve (AUROC). Other binary classification metrics such as Matthews correlation coefficient (MCC) resulted in the same parameters being selected. To measure model performance, both true positive and true negative sets are required. For SH3 interactions, true positive interactions were retrieved from Tonikian *et al.* [6] and true negative interactions were created using randomly selected interacting partners for each SH3 domains. The true negative set was constrained to exclude protein pairs annotated with overlapping cellular compartments, additionally true positive interactions were removed from the negative set. The parameter selected for the SH3 interaction network were the top scoring 30 interactions for each class, with accessibility and disorder scores respectively greater than 3 and 0 to achieve AUROC and MCC values of 0.86 and 0.79 respectively. For the kinase interaction network we used data from Breitkreutz *et al* [49]. Unfortunately, this dataset identifies only interaction partners and does not include interaction directionality, which is crucial for our purposes. To determine interaction directionality, *S. cerevisiae* phosphosite data from literature [18], [50]–[54] was superimposed on the Breitkreutz *et al* kinase network. Kinase PWMs from Mok *et al.* were subsequently used to identify kinase domains that bind specific phosphosites [11], [49]. To ensure the interaction is significant and likely a true positive, phosphoproteins were scanned by every kinase PWM to create a best score background distribution. For each kinase PWM best score distribution we assume it follows a Student's t-distribution and use a threshold of p-value<1×10^−16^ to define the true positive set. The kinase network true negative set was constructed in the same manner as the SH3 negative. Parameters selected for the kinase interaction network were top 360 interactions for each PWM class, accessibility score greater than 4.0 and a disorder score greater than 0.9 to achieve AUROC and MCC values of 1 and 0.16 respectively. In all comparisons, a 1 to 5 ratio between true positives and true negatives were used. Various metrics describing the confusion matrix were found to be similar across a range of true positive and true negative ratios (Figure S1).

### Network comparison and orthology assignment

Comparisons between the 23 different species interaction networks were made using orthology mappings provided by the SYNERGY algorithm [55]. Two network comparison types were performed, global and local, respectively based on the absence or use of exact amino acid positional target binding information. Global comparisons involve determining the interaction conservation between two proteins. Here an interaction is conserved if any of the target proteins has an ortholog found to also be a binding partner for a given protein domain (one-to-one and one-to-many orthology relationships). Local comparisons are used in identifying conserved interactions when estimating the network rewiring rate. To simplify network comparisons for evolutionary rate calculations, we constrained orthology mappings to one-to-one and many-to-one relationships with respect to the reference species, thus ensuring at most a single protein per species exists in a multiple sequence alignment for each reference species' protein. Orthology mappings are established using the shortest distance between genes, where the distances are derived from the orthogroup's reconstructed gene tree, as orthogroups encompass both orthologs and paralogs. The reconstructed gene trees for each orthogroup were retrieved from the January 2009 data revision by Wapinski I. *et al.* containing the 23 fungal species used in this study [25].

### Ortholog selection for *S. cerevisiae* proteins containing a SH3 or kinase domain

Blastp [56] and the SH3 Pfam HMMs [57] were used to determine SH3 domain existence in the orthologs to *S. cerevisiae* proteins containing a SH3 domain. If both blastp and the SH3 Pfam HMMs failed to identify a SH3 domain, the protein was removed from the interaction network. *S. cerevisiae* domain regions were retrieved from Tonikain *et al.* [6]. Parameters used for blastp were ‘-evalue 0.1’ and for the Pfam HMMs, ‘-e_seq 0.1 -e_dom 0.1’. In a similar manner, kinase proteins from Mok *et al.* were selected [11] and the Pfam Pkinase HMM was run with ‘-e_seq 0.01 -e_dom 0.01’. The *S. cerevisiae* kinase domain boundaries were extracted from the Pfam output, and blastp was run with ‘-evalue 0.1’. If both blastp and the Pkinase Pfam HMM failed to identify a kinase domain in an orthologous *S. cerevisiae* protein, the protein was removed from the kinase interaction network.

### Multiple sequence alignment

For each gene target involved in a SH3 or kinase interaction, the protein sequences of the gene target and its associated orthologs were aligned using MAFFT v6.717b [58] with the ‘–auto’ parameter.

### SH3, kinase, and canonical phylogenetic trees

The R statistical program with the Analyses of Phylogenetics and Evolution (APE) package [59] was used to generate the phylogenetic trees derived from conserved interactions in the SH3 and kinase interaction networks for the 23 yeast species using a minimum evolution method. As input for such methods, a multiple sequence alignment (MSA) is required. A MSA based on conserved interactions can be created by considering an interaction between a protein pair as a single position within a MSA, whose value is 1 if the interaction is present and 0 otherwise.

The canonical phylogenetic tree was created by concatenating the protein MSA of 79 out of 153 genes families with synteny support [27], [60], where the 79 gene families correspond to orthogroups containing each of the 23 yeast species exactly once [25]. In other words, only gene families with no paralogs were selected. MAFFT was used to align the 79 gene family orthogroups. The alignments were concatenated and a phylogenetic tree was created using SEMPHY version 2.0b3 with the JTT matrix and parameters ‘–jtt –S –O’ [61]. The divergence distance from the last common ancestor between *S. cerevisiae* and *S. pombe* was set to 600 million years and the APE package was used to create the canonical phylogenetic tree.

### Interaction change rates

To compare rates of interaction change with the computed SH3 and kinase interaction networks against prior literature rates, we used rates of interaction change provided by Beltrao and colleagues for the kinase and transcription factor networks. To provide a fair comparison, divergence times for *K. lactis*, *C. albicans*, and *S. pombe* with respect to *S.* cerevisiae were set to 300, 400, and 600 million years respectively, values use by Beltrao *et al.* [18]. Divergence times for the yeast sensu stricto group taken with respect to *S. cerevisiae* for *S. paradoxus*, *S. mikatae*, and *S. bayanus* were 10, 15, and 20 million years respectively [62].

Estimating rates of interaction change between interaction networks requires the divergence times between the networks to be known. To estimate divergence times, we created a canonical gene phylogenetic tree encompassing all 23 fungal species. The above section details the construction of the canonical phylogenetic tree.

The network rewiring mechanism of “interaction rewiring” and “protein change” require the identification of conserved interactions, as the absence of a conserved interaction is an interaction change. For interaction rewiring, an interaction is conserved if there exists a target binding site within a window centered on the reference species' target binding site plus 10 flanking amino acids. For protein change, interaction conservation is based solely on the existence of a protein ortholog.

Rates of interaction change with respect to a reference species were calculated in the same manner as Beltrao *et. al.* using the following equation:
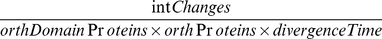
where *intChanges* is the number of gained and lost interactions, *orthDomainProteins* is the number of orthologous proteins between the two species in comparison containing a SH3 or kinase domain, *orthProteins* is the number of orthologous proteins, and *divergenceTime* is the divergence time in millions of years separating the two species [18]. Rates for both network rewiring mechanisms of interaction rewiring and protein change were calculated using the above equation. This equation can be viewed as the fraction of the interaction changes versus all possible interactions amongst proteins with orthologs and normalized by the divergence distance between the two compared species interaction networks.

Determining if rates of interaction rewiring significantly differ between the predicted network and random networks, 1000 sets of randomized interaction networks for each of the 23 species were created. The networks were randomized such that protein degree and the total number of nodes within the 23 interaction networks were maintained. For each set of randomized networks, the rate of interaction rewiring was calculated and compared against rates found in the original interaction networks.

Estimating the error in network rewiring was performed in the SH3 interaction network and between the two closest yeast species: *S.cerevisiae* and *S. paradoxus*. Using the predicted *S.cerevisiae* interaction network, the number of false positives and false negatives were calculated using the true positive and true negative datasets determined above. We assume the number of incorrect interactions is the same in the *S. paradoxus* SH3 interaction network, hence the maximum number of incorrect interactions is double the number of false interaction changes found in the *S.cerevisiae* network.

### Analysis of binding site clusters

The set of target binding sites, linear peptide sequences, for each SH3 and kinase domain were provided by the MOTIPS pipeline. When multiple target binding sites overlap, a target binding site cluster is formed. Specifically, a binding site cluster is defined as a region on a protein target for which every peptide segment bound by a protein domain overlaps with every other bound peptide segment, and for every pair of overlapping bound segments, one segment overlaps another by more than 50% of its peptide length. A greedy approach is used to form the clusters.

### Sequence conservation metrics

Determining the binding site sequence conservation is measured by AL2CO [36] in conjunction with a weighted scoring scheme to account for gaps. AL2CO was run with the parameters ‘–f 1 –g 0.01’. Each amino acid position within the multiple sequence alignment was further weighted by 1 minus the ratio of gaps vs non-gaps at that amino acid position, thereby decreasing the conservation score of positions with many gaps.

Calculating the sequence conservation specific to a protein domain is computed in 3 steps: 1) use PWMs to calculate the highest scoring target binding site for all protein orthologs at the location of the reference species' target binding site in a MSA within a 10 amino acid flanking window, 2) obtain divergence distances for all protein orthologs with respect to *S. cerevisiae*, and 3) finally weight the difference in PWM scores between protein orthologs versus the *S. cerevisiae* protein PWM score with the reciprocal of the protein orthologs divergence distance from the *S. cerevisiae* protein. Computing the highest scoring target binding sites for all protein orthologs for a given protein domain is performed by making a MSA for each target protein, where each MSA consists of the target protein and its protein orthologs. Using *S. cerevisiae* as the reference species, a window is set to the boundaries of the target binding site plus 10 flanking amino acids on either side. For each protein ortholog, the highest scoring target binding site is attained from the sequence within the window. A score threshold defined as twice the worst PWM score in the *S.cerevisiae* network is applied to the PWM score difference between protein orthologs and the *S. cerevisiae* protein to capture sequences likely to have diverged towards the *S. cerevisiae* binding target site. Divergence distances for protein orthologs of a *S. cerevisiae* target protein are obtained from the reconstructed gene tree by Wapinski I. *et al.* of the target protein's orthogroup [25]. If protein paralogs exist within the orthogroup, the protein with the shortest distance to the *S. cerevisiae* target protein is retrieved.

## Supporting Information

Figure S1 Effect of varying the true positive/true negative ratio on different metrics describing the confusion matrix for A) the SH3 interaction network and B) the kinase-substrate network.
(EPS)

Figure S2 Pairwise similarity of *S. cerevisiae* SH3 domain specificities against each other. A) PWM dissimilarity versus percent amino acid identity between two SH3 domains, where PWM dissimilarity is the normalized sum of the minimum L_2_ norm between two PWM positions by padding each PWM with flanking fake amino acid X. B) The number of common position specific interactions shared between two SH3 domains versus percent amino acid identity.
(EPS)

Figure S3 Multiple sequence alignment of *S. cerevisiae* SH3 domain paralogs and their orthologs for A) Boi1 and Boi2, B) Lsb1 and Pin3, C) Myo3 and Myo5, and D) Lsb3 and Lsb4. Many positions shared between orthologs are shared between paralogs. The MAFFT multiple sequence alignments [58] were visualized using JalView [63].
(EPS)

Figure S4 Contact amino acids, defined as amino acids 5 Å from a bound ligand in a crystal structure, for *S. cerevisiae* SH3 domains are highly similar to those found in their orthologs. Highlighted columns indicate contact amino acids for the *S. cerevisiae* SH3 domains in the multiple sequence alignment for A) Pex13 (2V1R and 1N5Z) [64], B) Sho1 (2VKN), C) Bbc1 (1ZUK), and D) Lsb3 (1SSH). PDB ids are in parenthesis. If two structures were present, the union of the contact positions was taken. The MAFFT multiple sequence alignments [58] were visualized using JalView [63].
(EPS)

Figure S5 SH3 domains in orthologous proteins exhibited a high degree of amino acid identity to their *S. cerevisiae* ortholog.
(EPS)

Figure S6 Kinase domains in orthologous proteins exhibited a high degree of amino acid identity to their *S. cerevisiae* ortholog.
(EPS)

Figure S7 Phylogenetic trees derived from A) SH3 interaction conservation and B) kinase interaction conservation for 23 fungal species.
(EPS)

Figure S8 Kinase rates of interaction change and number of kinase interaction changes. A) Rates of kinase interaction change calculated such that no branch is shared in the canonical phylogenetic tree versus divergence in millions of years. B) The number of kinase interaction changes with respect to *S. cerevisiae* versus divergence in millions of years.
(EPS)

Figure S9 A) SH3 interaction rewiring rates taken with respect to *S. cerevisiae* compared against rates taken from 1000 randomized networks. B) Enlargement of A, with a focus on more distance species to *S. cerevisiae*.
(EPS)

Figure S10 Rates of interaction change is similar when considering orthologs to *S. cerevisiae* A) SH3 and B) kinase proteins above various percent amino acid identity thresholds. Calculating the rates of interaction change for domains only used orthologous proteins meeting the threshold criteria.
(EPS)

Figure S11 Ranking the SH3 PWMs by their information content by summing the entropy at each position within the PWM fails to reveal a correlation between SH3 domains and the rate of interaction rewiring (ρ = −0.067, p-value = 0.724).
(EPS)

Figure S12 A strong correlation exists between interaction conservation and both binding cluster size and associated number of cluster on a protein in the *S. cerevisiae* kinase interaction network. A) A significant correlation between interaction conservation and binding cluster size exists (ρ = 0.210, p = 1.40×10^−14^) and B) interaction conservation and the number of clusters found on a protein (ρ = 0.296, p = 6.48×10^−10^).
(EPS)

Figure S13 Preferential binding of SH3 domains to the same GO functional groups across species. The Jensen-Shannon divergence was used to measure the distribution of GO terms of the target proteins bound by SH3 domains in other species with respect to their *S. cerevisiae* counterparts. A score of 0 indicates identity and 1 indicates complete difference in GO functional group distributions. Many species show a mean score of 0.15, indicating high similarity in GO functional groups bound by the SH3 domain in *S. cerevisiae*.
(EPS)

Table S1 Quantification of yeast SH3 interaction change due interaction rewiring. The number of interactions gained and lost is found in parenthesis. Rates after the backslash were calculated by using the closest species to the species in question from the gene derived phylogenetic tree.
(DOC)

Table S2 Quantification of yeast kinase interaction change due interaction rewiring. The number of interactions gained and lost is found in parenthesis. Rates after the backslash were calculated by using the closest species to the species in question from the gene derived phylogenetic tree.
(DOC)

Table S3 Quantification of yeast SH3 interaction change due protein change. The number of interactions gained and lost is found in parenthesis. Rates after the backslash were calculated by using the closest species to the species in question from the gene derived phylogenetic tree.
(DOC)

Table S4 Quantification of yeast kinase interaction change due protein change. The number of interactions gained and lost is found in parenthesis. Rates after the backslash were calculated by using the closest species to the species in question from the gene derived phylogenetic tree.
(DOC)

Table S5 Phosphoevolution rates adapted from Beltrao *et al.* to correspond to the ortholog mappings used in this study [18]. *S. cerevisiae* protein kinases were derived from Breitkreutz *et al.*, whose category was denoted as ‘kinase catalytic’. The corresponding orthologs were mapped to *C. albicans* and *S. pombe* (Methods) [49]. The range in rates is given by the assumption up to 5 interactions are either gained or lost following the gain or loss of a phosphoprotein.
(DOC)

Table S6 Transcription factor rates of interaction change with respect to *S. cerevisiae* are from Borenman *et al.* [19]. Rates were calculated in the same way as Beltrao *et al.* [18] but the number of orthologs and the divergence times are adjusted to reflect those used in this study.
(DOC)
