# Supplementary material for: Network Evolution: Rewiring and Signatures of Conservation in Signaling
Source: PLoS Comput Biol. 2012 Mar 15;8(3):e1002411. doi: 10.1371/journal.pcbi.1002411 (PMC3305342; doi:10.1371/journal.pcbi.1002411)
Supplement: Table S3 — Quantification of yeast SH3 interaction change due protein change. The number of interactions gained and lost is found in parenthesis. Rates after the backslash were calculated by using the closest species to the species in question from the gene derived phylogenetic tree. (DOC) [file pcbi.1002411.s016.doc]

| **Species** | **SH3 domain orthologs** | **Orthologs** | **Changed interactions**  **total (gain,loss)** | **Divergence Time (My)** | **SH3 interaction change (per protein pair per My)** |
| --- | --- | --- | --- | --- | --- |
| *S. paradoxus* | 21 | 5096 | 61 (34,27) | 10 | 5.70x10^-5^ / 4.74x10^-5^ |
| *S. mikatae* | 21 | 4913 | 88 (23,65) | 15 | 5.69x10^-5^ / 4.08x10^-5^ |
| *S. bayanus* | 21 | 4996 | 91 (53,38) | 20 | 4.34x10^-5^ / 2.68x10^-5^ |
| *C. glabrata* | 18 | 4649 | 82 (54,28) | 124 | 6.58x10^-6^ / 6.58x10^-6^ |
| *S. castelli* | 17 | 4688 | 43 (19,24) | 149 | 4.34x10^-6^ / 4.34x10^-6^ |
| *K.waltii* | 17 | 4358 | 109 (24,85) | 191 | 7.70x10^-6^ / 2.62x10^-5^ |
| *K. lactis* | 17 | 4431 | 56 (9,47) | 191 | 3.89x10^-6^ / 7.24x10^-6^ |
| *S. kluyveri* | 17 | 4333 | 172 (45,127) | 191 | 1.22x10^-5^ / 2.62x10^-5^ |
| *A. gossypii* | 17 | 4311 | 115 (59,56) | 191 | 8.22x10^-6^ / 1.18x10^-5^ |
| *C. lusitaniae* | 16 | 3803 | 355 (172,183) | 191 | 1.46x10^-5^ / 1.16x10^-5^ |
| *D. hansenii* | 17 | 3969 | 308 (139,169) | 400 | 1.14x10^-5^ / 9.90x10^-6^ |
| *C. guilliermondii* | 17 | 3814 | 329 (151,178) | 400 | 1.27x10^-5^ / 9.90x10^-6^ |
| *C. tropicalis* | 16 | 3858 | 418 (165,253) | 400 | 1.69x10^-5^ / 1.92x10^-5^ |
| *C. albicans* | 17 | 3982 | 381 (137,244) | 400 | 1.41x10^-5^ / 1.92x10^-5^ |
| *C. parapsilosis* | 17 | 3812 | 332 (149,183) | 400 | 1.28x10^-5^ / 1.26x10^-5^ |
| *L. elongosporus* | 17 | 3805 | 361 (155,206) | 400 | 1.40x10^-5^ / 1.33x10^-5^ |
| *Y. lipolytica* | 15 | 3616 | 579 (222,357) | 442 | 2.41x10^-5^ / 2.41x10^-5^ |
| *A. nidulans* | 14 | 3351 | 764 (349,415) | 519 | 3.14x10^-5^ / 3.28x10^-5^ |
| *N. crassa* | 14 | 3337 | 838 (380,458) | 519 | 3.46x10^-5^ / 3.28x10^-5^ |
| *S. japonicus* | 15 | 3132 | 464 (279,185) | 600 | 1.65x10^-5^ / 8.38x10^-6^ |
| *S. octosporus* | 15 | 3219 | 430 (271,159) | 600 | 1.48x10^-5^ / 4.14x10^-6^ |
| *S. pombe* | 15 | 3247 | 426 (271,155) | 600 | 1.46x10^-5^ / 4.42x10^-6^ |
| **Average** |  |  |  |  | **1.97x10^-5^ / 1.95x10^-5^** |
