# Supplementary material for: Network Evolution: Rewiring and Signatures of Conservation in Signaling
Source: PLoS Comput Biol. 2012 Mar 15;8(3):e1002411. doi: 10.1371/journal.pcbi.1002411 (PMC3305342; doi:10.1371/journal.pcbi.1002411)
Supplement: Table S5 — Phosphoevolution rates adapted from Beltrao et al. to correspond to the ortholog mappings used in this study [18]. S. cerevisiae protein kinases were derived from Breitkreutz et al., whose category was denoted as ‘kinase catalytic’. The corresponding orthologs were mapped to C. albicans and S. pombe (Methods) [49]. The range in rates is given by the assumption up to 5 interactions are either gained or lost following the gain or loss of a phosphoprotein. (DOC) [file pcbi.1002411.s018.doc]

| **Species** | **Orthologous Kinases** | **Orthologs** | **Diverged Phospho-proteins** | **Divergence Time (My)** | **Interaction changes (per protein pair per My)** |
| --- | --- | --- | --- | --- | --- |
| *C. albicans* | 93 | 3982 | 322 | 400 | 2.17x10^-6^ to 1.09x10^-5^ |
| *S. pombe* | 78 | 3247 | 377 | 600 | 2.48x10^-6^ to 1.24x10^-5^ |
| **Average** |  |  |  |  | 2.33x10^-6^ to 1.17x10^-5^ |
