# Supplementary material for: Network Evolution: Rewiring and Signatures of Conservation in Signaling
Source: PLoS Comput Biol. 2012 Mar 15;8(3):e1002411. doi: 10.1371/journal.pcbi.1002411 (PMC3305342; doi:10.1371/journal.pcbi.1002411)
Supplement: Table S6 — Transcription factor rates of interaction change with respect to S. cerevisiae are from Borenman et al. [19]. Rates were calculated in the same way as Beltrao et al. [18] but the number of orthologs and the divergence times are adjusted to reflect those used in this study. (DOC) [file pcbi.1002411.s019.doc]

| **Species** | **Transcription Factor** | **Orthologs** | **Changed interactions** | **Divergence Time (My)** | **Interaction changes (per protein pair per My)** |
| --- | --- | --- | --- | --- | --- |
| *S. bayanus* | Tec1 | 4996 | 40 | 20 | 4.00x10^-4^ |
| *S. mikatae* | Tec1 | 4913 | 96 | 15 | 1.30x10^-3^ |
| *S. bayanus* | Ste12 | 4996 | 78 | 20 | 7.81x10^-4^ |
| *S. mikatae* | Ste12 | 4913 | 54 | 15 | 7.33x10^-4^ |
| **Average** |  |  |  |  | 8.04x10^-4^ |
